# Whole exome sequencing links dental tumor to an autosomal-dominant mutation in *ANO5* gene associated with gnathodiaphyseal dysplasia and muscle dystrophies

**DOI:** 10.1038/srep26440

**Published:** 2016-05-24

**Authors:** T. V. Andreeva, T. V. Tyazhelova, V. N. Rykalina, F. E. Gusev, A. Yu. Goltsov, O. I. Zolotareva, M. P. Aliseichik, T. A. Borodina, A. P. Grigorenko, D. A. Reshetov, E. K. Ginter, S. S. Amelina, R. A. Zinchenko, E. I. Rogaev

**Affiliations:** 1 Department of Genomics and Human Genetics, Laboratory of Evolutionary Genomics, Vavilov Institute of General Genetics, Russian Academy of Sciences, Moscow 119991, Russia; 2 Center for Brain Neurobiology and Neurogenetics, Institute of Cytology and Genetics, Siberian Branch of the Russian Academy of Sciences, Novosibirsk 630090, Russia; 3 Max-Planck Institute for Molecular Genetics, Berlin 14195, Germany; 4 Alacris Theranostics GmbH, Berlin 14195, Germany; 5 Freie Universitaät Berlin, Berlin 14195, Germany; 6 Faculty of Bioengineering and Bioinformatics, Center of Genetics and Genetic Technologies, Lomonosov Moscow State University, Moscow 119234, Russia; 7 Department of Psychiatry, Brudnick Neuropsychiatric Research Institute, University of Massachusetts Medical School, Worcester, Massachusetts 01604, USA; 8 Federal State Budgetary Institution «Research Centre for Medical Genetics», Moscow 115478, Russia; 9 The Rostov State Medical University, Rostov-on-Don 344022, Russia; 10 Pirogov Russian National Research Medical University, Moscow 117997, Russia

## Abstract

Tumors of the jaws may represent different human disorders and frequently associate with pathologic bone fractures. In this report, we analyzed two affected siblings from a family of Russian origin, with a history of dental tumors of the jaws, in correspondence to original clinical diagnosis of cementoma consistent with gigantiform cementoma (GC, OMIM: 137575). Whole exome sequencing revealed the heterozygous missense mutation c.1067G > A (p.Cys356Tyr) in *ANO5* gene in these patients. To date, autosomal-dominant mutations have been described in the *ANO5* gene for gnathodiaphyseal dysplasia (GDD, OMIM: 166260), and multiple recessive mutations have been described in the gene for muscle dystrophies (OMIM: 613319, 611307); the same amino acid (Cys) at the position 356 is mutated in GDD. These genetic data and similar clinical phenotypes demonstrate that the GC and GDD likely represent the same type of bone pathology. Our data illustrate the significance of mutations in single amino-acid position for particular bone tissue pathology. Modifying role of genetic variations in another gene on the severity of the monogenic trait pathology is also suggested. Finally, we propose the model explaining the tissue-specific manifestation of clinically distant bone and muscle diseases linked to mutations in one gene.

Cemento-ossifying fibromas of the mandible and/or maxilla are the characteristic lesions of autosomal dominant gnathodiaphyseal dysplasia (GDD, OMIM: **166260**), which involves fibro-osseous lesions of the jawbones with a complex skeletal syndrome of bone fragility, bowing/cortical thickening of tubular bones, and diaphyseal sclerosis of long bones^1^,^2^. Gigantiform cementoma (GC, OMIM: 137575) is a rare form of dental tumor that can be associated with bone fractures. It is inherited as an autosomal dominant trait and shows variability in clinical manifestation^3^,^4^,^5^,^6^.

GDD is one of a pathological spectrum of generalized skeletal syndromes, which are characterized by cemento-osseous lesions of the jawbones including fibrosis dysplasia (FD), cemento-ossifying fibroma (COF) and McCune–Albright syndrome (MAS)^7^,^8^. GDD can be differentiated from FD and MAS on the basis of distinctive clinical, histological, and molecular features. Repeated fractures caused by minor accidents in childhood and adolescence are common in both FD and GDD, although these two can be differentiated radiographically by the presence of diaphyseal cortical thickening and bowing, which are seen exclusively in GDD. In GDD, fractures heal within the normal process and with no symptoms of pseudarthrosis or bone deformity, and patients are characterized by a normal stature. In addition, GDD is not associated with significant changes in markers of bone metabolism^6^,^9^. The clinical picture of GDD does not include skin pigmentation or endocrine malfunction, thus distinguishing it from MAS. In familial cases of GC, described up till now, some affected family members have bone fractures and share the clinical symptoms with GDD, but some affected individuals with GC do not have a history of the bone fractures^1^,^2^,^3^.

It has been shown recently that three mutations in the anoctamine 5 gene (*ANO5*) are responsible for the GDD in an African-American, Japanese, and Italian families^10^,^11^. Mutation in the type I collagen gene (*COL1A1*) was found to be associated with multiple fractures and fibro-osseous lesions in the jaw^12^. Currently no other mutations in patients with GC or GDD have been identified.

Anoctamin 5 belongs to the anoctamin protein family. All 10 human anoctamins (ANO1-ANO10) were shown to be Ca^2+^-activated proteins with either putative channel or scramblase or both channel and scramblase functions^13^. Members of the anoctamin family contain several transmembrane domains. To date, X-Ray analysis of fungal anoctamin homolog revised the number of membrane-spanning domains to ten^14^. The molecular function of ANO5 is presently unknown. The functional analysis of ANO5 fail to find it as plasma membrane Ca^2+^-activated chloride channel activity, the function described for the anoctamins ANO1 and ANO2^11^.

In this study, we will present the genetic analysis of Russian pedigree with familial dental tumor and severe bone deformities of both legs in two brothers originally diagnosed with cementoma and suggested gnathodiaphyseal dysplasia^9^, as well as probands’ mother with cementoma and healthy probands’ father.

## Results

**Figure 1:**
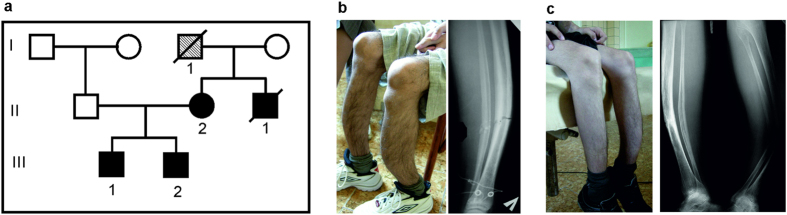
Family examined in this study. (**a**) Family with cementoma and repeated bone fractures. (**b,c**) Clinical phenotypes of two brothers (III-1 and III-2) include fragility and deformation of tubular bone. The detailed clinical descriptions of facial tumors overlapped with familial gigantiform cementoma (OMIM: 137575) were made previously^9^. The affected siblings and their unaffected father were used for genetic analysis. The mother (II-2) that was unavailable for the study had an operation on cementoma lower jaw, but had no GDD clinical diagnosis in her medical history. No limbs deformation were expressed in the mother. The probands’ uncle (II-1) and grandfather (I-1) had repeated bone fractures (see. *Material and Methods*).

**Figure 2:**
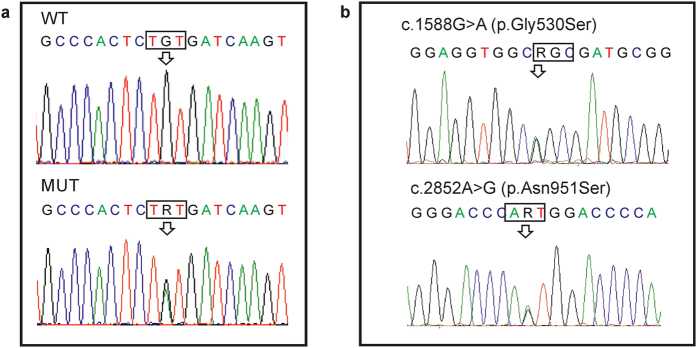
Genetic analysis of the family. (**a**) Sanger sequence chromatograms of *ANO5* are shown for one affected individual (MUT) and unaffected father (WT). The arrow indicates the heterozygous mutation at the position c.1067G > A (p.Cys356Tyr) of *ANO5* gene in the affected individual. (**b**) Genomic sequence chromatograms of *COL5A1* identified two heterozygous mutations in the *COL5A1* gene (designated by arrows) in the affected brothers, which were inherited from their father (shown on the chromatogram).

The whole-exome sequencing was performed on the two affected individuals (Fig. 1). Approximately 81 and 47 million reads were generated for each individual. The percentage of reads mapped to reference human genome hg19 was 96.1% and 98.7%, resulting in a mean exome coverage of ×43.8 and ×26.1 (Table 1). Using the filtering strategy, described in the *Data processing* section, we identified heterozygous mutation in the anoctamin 5 gene in both affected individuals (Fig. 2). This mutation is located in exon 11 of *ANO5* gene (c.1067G > A) and causes the substitution of cysteine in position 356 with tyrosine (p.Cys356Tyr).

This mutation has not been reported in dbSNP138, the 1000 Genomes databases, ClinVar records, or in our own data set of whole genome sequences from the same ethnic group (Russian origin). Sanger sequencing confirmed the presence of the variant in both affected brothers but not in their unaffected father (Fig. 2). Genetic analysis of the mother was not performed due to her refusal to provide the biological material.

**Figure 3:**
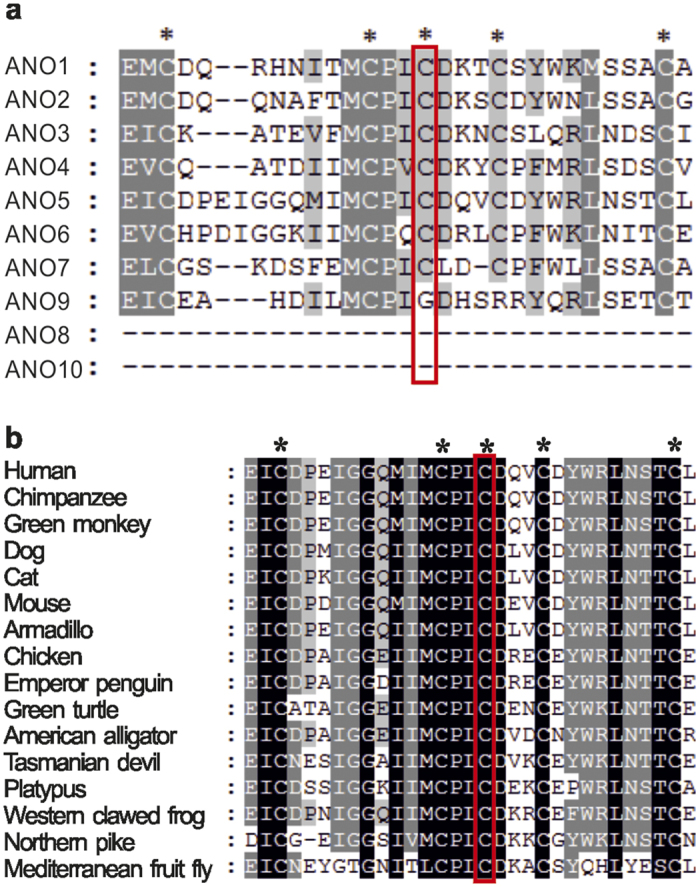
Amino-acid sequence alignment of ANO5 with (**a**) paralogous human proteins and (**b**) orthologous proteins. Conserved cysteine residues are marked by asterisks and the p.356Cys position of ANO5 is boxed.

The variant was predicted to be probably damaging by PolyPhen2 tool and damaging by SIFT tool. The cysteine residue at position p.356Cys of *ANO5* gene is highly conserved across orthologous genes of various species, as well as in human paralogous of the anoctamin 5 gene^10^ (Fig. 3).

Additionally, two rare heterozygote mutations were found in the collagen gene *COL5A1* in both affected probands: c.1588G > A (p.Gly530Ser) and c.2852A > G (p.Asn951Ser). The two mutations are located on the same allele inherited by two affected siblings from their father (Fig. 2b). The mutation of the collagen gene *COL5A1* (p.Asn951Ser) had previously been found to cause Ehlers-Danlos syndrome in homozygote- or compound heterozygote- carriers^15^,^16^. Mutations in another collagen gene *COL1A1* were described to be associated with Ehlers-Danlos syndrome, characterized by osteogenesis imperfecta, bone fragility and other GDD-related lesions^12^,^17^. We then analyzed the 1000 Genomes project database, which currently include about 2500 individuals, and confirmed the genetic linkage of these two rare *COL5A1* mutations. Among 34 carriers of the c.1588G > A p.Asn951Ser (MAF = 0.0068) mutation, identified in the database, only 6 individuals do not have the second mutation c.2852A > G p.Gly530Ser (MAF = 0.02).

**Figure 4:**
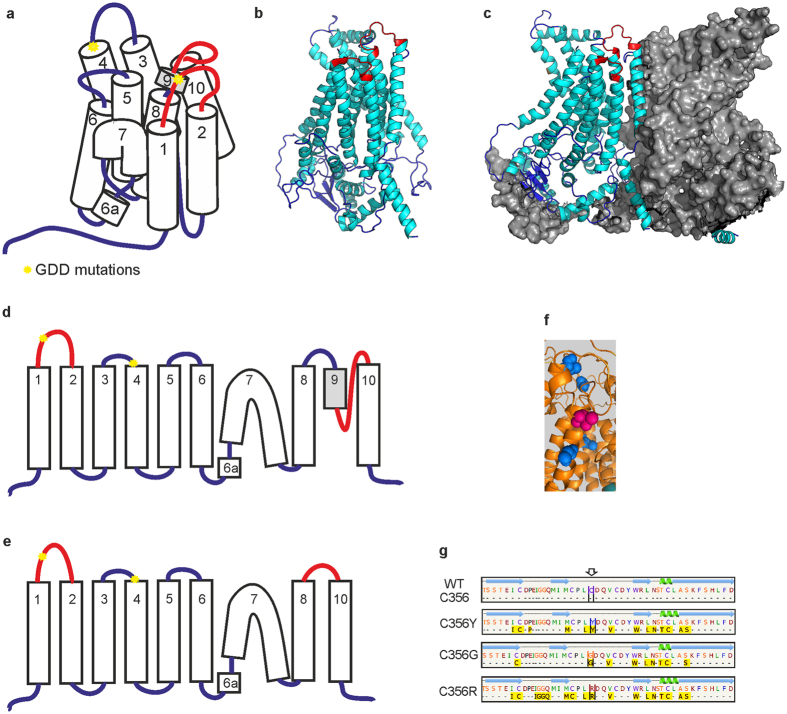
Predicted structure and helices topology of wild-type and mutant human ANO5 protein based on Phyre2 prediction. (**a,d**) Transmembrane domains in wild type ANO5. (**b**) Monomer ANO5 structure. (**c**) ANO5 subunit in dimer structure. Both loops with p.Cys356 and the area which change its predicted structure (loss of TMD predicted structure) are marked by red. (**e**) p.Cys356 mutations change the structure of region between 8 and 10 TMD. (**f**) Cysteine residuaes p.Cys520, p.Cys572, p.Cys601, p.Cys606 (blue) is located close to p.Thr513 (magenta) in predicted protein structure. (**g**) The largest pockets were detected by the fpocket2 program^44^^,^^45^ in the loop between 1^st^ and 2^nd^ TMDs of ANO5. Green helixes are alpha helixes, blue arrows are beta strands. Yellow boxes indicate the amino acids involved in the pocket formation. No large pockets were predicted for wild-type protein structure.

Three of the four GDD mutations detected in *ANO5* gene cause aminoacid changes at evolutionary conserved codon p.Cys356 (Fig. 3). We analyzed the predicted effects on the secondary structure of the ANO5 protein variant p.Cys356Tyr and previously reported GDD mutations p.Cys356Gly, p.Cys356Arg and p.Thr513Ile by Phyre2 server^18^. The transmembrane helices prediction based on recently published X-ray structure of TMEM scramblase (PDB ID:4WIT)^14^ revealed ten membrane-spanning transmembrane domains (TMD) in human ANO5 protein in contrast to eight TM structure previously predicted by hydropathy analysis^10^. All three known GDD mutations, which substitute cysteine in position p.356Cys, change the number of predicted TMDs to nine in the mutant ANO5 protein (Fig. 4d,e). Furthermore, the loop, containing p.Cys356 in predicted protein structure lies in close proximity to the transmembrane helix 9. The transmembrane helix 9 may be involved into dimer or dimer pocket formation due to its location close to the ninth TMD of the second subunit in dimer structure (Fig. 4c).

A closer analysis of the loop containing p.Cys356 revealed a candidate pockets in extracellular loop of ANO5 by the fpocket2 program for p.Cys356Tyr, p.Cys356Gly and p.Cys356Arg mutations. No pockets in wild-type extracellular loop were predicted (Fig. 4g).

**Figure 5:**
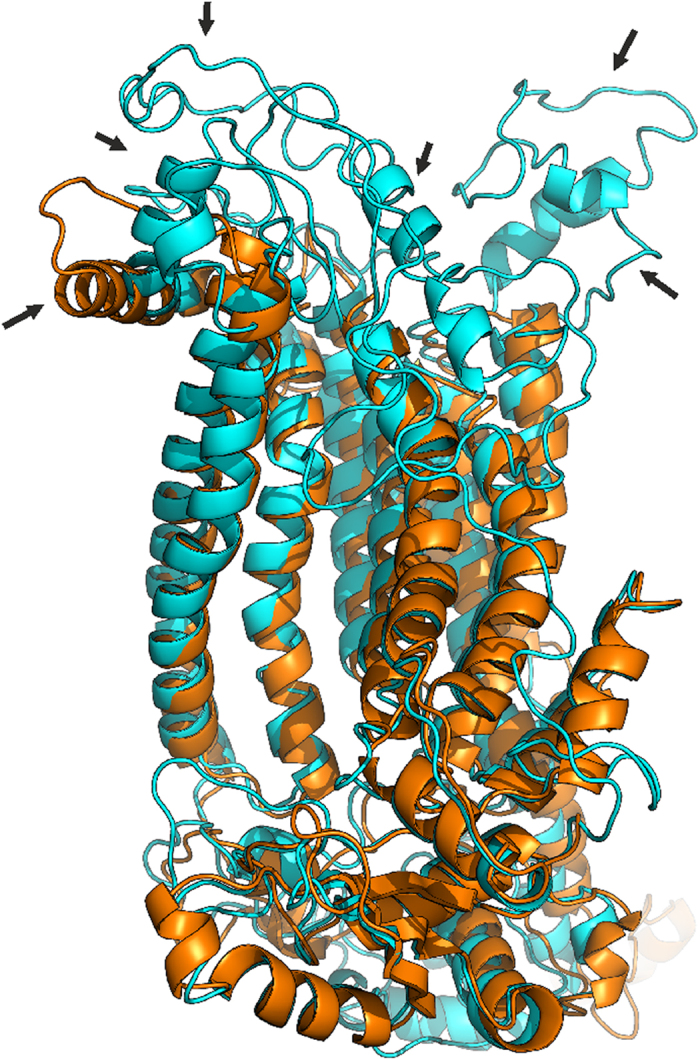
3D alignment of predicted structures of the wild-type ANO5 protein (cyan) and p.Ile513 mutant form (gold) revealed the differences in the extracellular loops (indicated by arrows).

Recently, a mutation in the fourth transmembrane domain of *ANO5* gene (c.1538C4T p.Thr513Ile) has been identified in Italian pedigree with GDD^11^. The p.Thr513Ile mutation does not change the predicted number of TMs. At the same time, several cysteine residuels surround the p.Thr513 position in the protein structure (p.Cys520, p.Cys572, p.Cys601, p.Cys606, Fig. 4f) and both p.Cys601 and p.CysC606 are faced to the same extracellular domain as p.Сys356. By using Phyre2, we revealed that p.Thr513Ile mutation may affect the predicted structure of the protein by altering the structure of several extracellular loops of ANO5 (Fig. 5).

## Discussion

A heterozygous mutation in the anoctamin 5 gene c.1067G > A (p.Cys356Tyr) was identified in both affected individuals in the Russian family with giant cementoma and bone fractures. Mutations in the same amino acid position (c.1066T > G p.Cys356Gly and c.1066T > A p.Cys356Arg) have been previously reported to be responsible for the gnathodiaphyseal dysplasia in an African-American and Japanese families^10^. The only other mutation in another codon of *ANO5* gene (c.1538C > Tp.Thr513Ile) has been reported to segregate with bone pathology in a large Italian GDD family^11^.

The original diagnosis for our patients was made and recorded in medical history based on clinical characteristics of dental tumor malformations and other symptoms completely consistent with giant cementoma (OMIM: 137575, *Material and Methods*). These types of clinical pathology match also GDD (OMIM: 166260). The clinical phenotype characteristics of our patients with GC are similar to those described for patients with mutations in the codon p.356 of ANO5 and include fibro-osseous facial tumors as well as long bone bowing with cortical thickening. Our results confirm that the patients initially diagnosed as GC have clinical and genetic manifestation defined now as GDD^9^. When this manuscript was in preparation, the letter was published indicating clinical laboratory report for the similar mutation in patients initially diagnosed with polyostotic fibrous dysplasia, which lesions overlapped with GDD^19^. Taken together, these data suggest that all three diseases (GC, polyostotic fibrous dysplasia and GDD) are likely the same or overlapped types of bone pathology.

In this study, we found two rare heterozygous variants in the collagen gene *COL5A1* in affected siblings. Mutations in this gene are associated with classic Ehlers-Danlos syndrome^20^. Another collagen gene, *COL1A1*, was found to be associated with specific fibro-osseous lesions in the skull and jaw, as well as osteogenesis imperfecta and bone fragility^12^. The variant alleles occur with relatively low population frequencies (MAF = 0.02 for rs61735045 and MAF = 0.0068 for rs61736966 in 1000 Genomes database). These two rare variants are linked in *cis*-position of *COL5A1* gene and are inherited by the affected siblings as a single father’s allele. Thus, we can assume that these variants are not present in their mother. The manifestation of the disease is supposed to be more severe in both siblings than in the mother and in the affected mother’s relatives (Fig. 1, see. *Material and Methods*). It should be noted, that some intrafamilial variability were described within the affected members in recently published GDD families. For example, the early age of the first fracture and thickening of tubular bones were not present in all carriers of the ANO5 mutation in two families^11^,^19^. Therefore, it is possible other genes can modify the manifestation of the pathology. The rare *COL5A1* allele inherited from father in the Russian pedigree could potentially contribute to the severity of the phenotype caused by *ANO5* dominant mutation, but further studies must be performed to confirm this suggestion.

Presently it is known that three of four dominant bone pathology mutations are located in the loop between the first and second TMD of ANO5 protein. As we have shown (Fig. 4b,c), this region neighbors the cavity formed by two ANO5 subunits in dimer, but the role of this region in protein function is currently unclear. Additionally, our protein structure prediction indicates, that mutations in codon p.Cys356 may disturb the last TMD of ANO5 protein, which has been found to be directly involved into dimer and dimer cavity formation^14^. Therefore, we can suggest, that mutations in the highly conserved p.Cys356 as well as amino acid change in p.Thr513 codon disturb dimer formation, perhaps through disruption cysteine of bridges involved in the process. The destruction of the disulfide bond may cause the formation of a pocket in the extracellular loop of the mutant forms of the ANO5 protein (Fig. 4g). As a result, some unknown ligands bind to the protein significantly altering its structure. Therefore the subunit cavity becomes destroyed and unable to perform its normal, yet unknown function.

Previously, *ANO5* gene mutations were found to be responsible for autosomal recessive muscular dystrophies, e.g., Limb girdle muscular dystrophy type 2L (LGMD2L, MIM:611307) and Miyoshi-like myopathy (MMD3, MIM:613319). Most of the muscular dystrophy mutations in *ANO5* gene are homozygotes or compound heterozygotes and lead to ANO5 deficiency due to frameshift or truncation of the protein or splice site changes representing loss-of-function phenotype^21^,^22^. These *ANO5* mutations spread across the gene, indicating the absence of mutation hot spots in muscular dystrophy patients.

In contrast, mutations in two ANO5 codons cosegregate with autosomal dominant pathogenic phenotype related to the bone, but not to the muscle tissue. Our data, along with previous reports, demonstrate the significance of mutations in single amino-acid position p.Cys356 for particular bone tissue pathology.

There is only one common lesion for bone pathology and muscular dystrophy related to *ANO5* gene mutations: both types of diseases include pathological fatty features in bone tissue site and muscle tissue sites for GDD and muscular dystrophy respectively^6^,^9^,^23^. According to TMEM16 X-Ray analysis, the dimer cavity contains lipids^14^, which can indicate its involvement in cell lipid metabolism. On this evidence, ANO5 dimer dysfunction may be specific not only for GDD mutation, but also for the pathologic activity in case of muscular dystrophy mutations. Most of muscular dystrophy mutations are frameshift, or caused by truncation of the protein, or splice site changes, and thus mutant ANO5 protein could not form functional dimer structures. In this way, the lipid metabolism of cells become broken, and the abnormal accumulation of phospholipids in the affected tissue can be detected in the form of psammomatoid bodies in bone tissue, up to complete replacement of muscles by fat in case of muscular dystrophy.

*ANO5* expression level in humans is high only in skeletal and heart muscle tissue, not in osteoblasts^10^,^24^, which may indicate instability and rapid degradation of ANO5 in the cell, in particular mutant forms of the protein^25^. The high level of expression of *ANO5* in human bone tissue is observed in pathologic condition like osteosarcoma (SaOS-2, U2OS cell lines and others)^26^,^27^. The different patterns of *ANO5* expression in the bone and muscle cells during the embryonic development^24^ and cell differentiation^27^ may be one of the explanations why the loss-of-function recessive mutations in *ANO5* lead to muscular dystrophy phenotype but do not affect the bone tissue.

Notably, ANO6 has the highest sequence homology to ANO5 among all anoctamine family proteins^13^,^28^ (Fig. 3a). The ANO6 operates as a membrane phospholipid scramblase^29^,^30^. It is highly expressed in mature bone tissue and it has been reported as an essential protein required for proper bone mineralization by activating phosphatidylserine scrambling in osteoblasts^31^,^32^,^33^. Both ANO5 and ANO6 proteins show similar intracellular localization^28^,^34^ and therefore may share structural and functional similarity. We speculate that the functional activities of ANO5 and ANO6 are redundant, as ANO6 is highly expressed in osteoblasts and its inactivation leads to demineralization of bones and skeletal deformities in mice^31^. Therefore, presumably ANO6 activity can compensate in the bone tissue for the loss-of-function of ANO5, caused by recessive mutations in clinical cases with muscle dystrophies.

We next hypothesize that gain-of-function mutation in ANO5 may cause bone pathology via interaction of ANO5 and ANO6 molecules. The ANO proteins may form homodimers and heterodimers^14^,^34^,^35^,^36^ and ANO6 was shown to oligomerize with another human anoctamin ANO2^37^. Therefore, it is possible that the highly homologous ANO5 and ANO6 are also capable of forming heterodimeric structures.

Based on recently resolved TMEM16 dimer structure^14^, we suggest two distinct mechanisms/functions of ANO5 in skeletal and muscle tissues. One may be related to individual subunits’ activity and be required in muscle cells, while the other deals with dimers activity essential in the bone, but not the muscle tissues. The differential tissue expression of *ANO5* and *ANO6* genes and functional dichotomy of ANO5 occurring in active monomeric and dimeric forms may potentially explain the pathogenic effect of gain-of-function mutation of *ANO5* gene in bone tissue, but not in muscle tissue. Nevertheless, further studies will be needed to resolve these hypotheses and the role of ANO5 in a variety of human pathologies.

## Materials and Methods

### Clinical description and Samples

Two siblings of Russian origin from the Rostov region were originally clinically diagnosed with facial tumor. Both probands had a similar clinical picture with familial gigantiform cementoma at theirs 8–10 years old, as well as severe deformities and multiple fractures of both legs, radiuses and ulnas, which have been developed later and occurred under light load conditions. A subtotal mandibulectomies with mandibular prosthesis reconstruction as well as surgical treatment of maxillary tumors were performed for the siblings. Detailed clinical description of probands presenting the facial tumors overlapped with familial GC (OMIM: 137575) was done by Roginsky^9^. Specifically, clinical features of jaws tumor in both brothers showed protuberant «Akhenaten»-type mandible consistent with GC. As previously suggested, Akhenaten, who was the father of ancient Egypt King Tutankhamen, may had gigantiform cementoma^2^. The X-ray and CT scans of the jaw in younger brother (III-2) showed overgrowth in the mandibular and maxillary bones, and fibro-osseous lesions with psammomatoid bodies^9^. Independently, we evaluated the family history and inheritance of this pathology (Fig. 1). According to the medical records, the clinical diagnosis of cementoma was made for the mother (II-2) based on her anamnesis and vitae and physical examination. She had a prosthetic lower jaw, as a result of reconstructive surgery due to cementoma of the low jaw. No limbs deformations were expressed in the mother or in other members of her family. The mother moves by herself without assistance. The uncle (II-1) and grandfather (I-1) of the probands of the maternal lineage had frequent bone fractures and the uncle has been diagnosed with congenital bone fragility (Fig. 1). By the time of our inspection, the elder brother (III-1) moves around with assistance, using an orthopedic cane. He had 12 pathological fractures. The younger brother (III-2) moves only in a wheelchair. By the time of the inspection, he had 14 pathological bone fractures, the latest of which occurred while he attempted to stand up from the wheelchair. He has severe deformations of the bones in both shins.

The study protocols were approved by the local Ethics Committee of Research Centre for Medical Genetics with the regulations and guidelines outlined in the Declaration of Helsinki. The experiment methods were carried out in accordance with the approved guidelines. Written informed consents were obtained from all participants of the study.

Genomic DNA was extracted from peripheral blood leukocytes by standard phenol-chloroform method. DNA integrity was tested by gel electrophoresis and the DNA concentration was determined using dsDNA BR kit (Invitrogen, Q32853) and Qubit fluorimeter. Extracted DNA samples were used for whole-exome and Sanger sequencing experiments.

### Whole-Exome Sequencing

The whole-exome library preparation procedure was performed as described previously^38^. In brief, whole genome amplification (WGA) was performed using REPLI-g Mini Kit (Qiagen) and followed by standard steps for Illumina DNA library preparation using the SureSelect^XT2^ Reagent kit (Agilent, Cat. No. G9621A). The resulting libraries were subjected to exome enrichment using the Agilent SureSelect^*XT2*^ Human All Exon v4 + UTRs capture probes set (Agilent, Cat. No. 5190-4671) following manufacturer’s instructions. In procedure of exome enrichment, the cementoma patients’ libraries were pooled with other human genomic libraries.

Hybridization of pooled libraries to the capture probes and removal of non-hybridized library molecules were carried out according to the Agilent SureSelect^XT2^ Target Enrichment System for Illumina Multiplexed Sequencing Protocol (version B, April 2012), with amplification of the final library. To avoid excessive amplification the copy number of the library was determined first by qPCR by using 1 μl of the captured library. An optimal PCR cycles number adjusted to the volume of the library to avoid PCR plateau, was determined based on the amplification plot and was found to be 11.

Prepared whole-exome libraries were sequenced on an Illumina HiSeq2000 platform as paired-end 101-bp reads.

### Data processing

BWA tools version 0.5.9^39^ were used to align the generated reads to human reference genome assemble build hg19 GRCh37 (http://genome.ucsc.edu/). PCR duplicates were removed from alignment reads with Picard toolkit (http://broadinstitute.github.io/picard). SNPs and indels were predicted using GATK pipeline^40^. Variants were annotated using Variant Effects Predictor^41^.

Candidate variations were selected by comparing all the genetic variations identified in the exome data in this family to variations in genome sequences for 2504 individuals (1000 genomes project phase 3 release) and to our own human genome sequence datasets for 23 individuals of Russian origin. The SNPs and short indels with MAF <5% were selected, the synonymous SNPs were removed and all the protein-altering variants (non-synonymous SNPs and indels) were used for further analysis. PolyPhen2 and SIFT^42^,^43^ were used to predict the functional effects of candidate mutations.

The raw read sequences are available through the NCBI Sequence Read Archive (PRJNA295503).

Data processing and analysis were carried out by workflow pipeline designed in our laboratory (http://rogaevlab.ru/ngs-pipeline).

### Mutation Validation Analysis

Targeted re-sequencing of candidate variations was performed by polymerase chain reaction (PCR) of target region followed by DNA sequencing. The target region of the *ANO5* gene (GenBank accession number NM_213599) including the p.356 codon was amplified using primer pair ANO_F (5′-CTTGATTGCCCCTTCTGTTA-3′) and ANO_R (5′-ACCCCAAATTCCCATGAATA). Primer sequences for *COL5A1* gene mutations verification were COL5_1F (5′-GATCCAAACCAGACCCTCT-3′), COL5_1R (5′-TCTGAGCCTGACTCACTCAC-3′), COL5_2F (5′-GTGGGAAGAAATGACACCTG-3′) and COL5_2R (5′-GGGCACTAACACAGAACTCA-3′). Direct sequencing of target regions was performed using the BigDye Terminator Cycle Sequencing Ready Reaction Kit, v. 3.1 (Applied Biosystems, Foster, CA), and analyzed with an ABI Prism 3730XL Genetic Analyzer.

Possible protein conformations of normal and mutant ANO5 proteins were generated using Phyre server^18^.

## Additional Information

**How to cite this article**: Andreeva, T. V. *et al*. Whole exome sequencing links dental tumor to an autosomal-dominant mutation in *ANO5* gene associated with gnathodiaphyseal dysplasia and muscle dystrophies. *Sci. Rep.* **6**, 26440; doi: 10.1038/srep26440 (2016).

## Figures and Tables

**Table 1:** Exome sequencing statistics.

Patient	Sample name	Number of raw reads	Percentage of reads mapped to hg19	Mean exome coverage	Total polymorphic sites	Protein-altering variants* (MAF** < 0.05)
III-1	S000094	81220550	96.1	43.8	268898	2844
III-2	S000256	46949678	98.7	26.1	240488	1853

^*^Variants with HIGH or MODERATE impact on canonical protein isoforms.

^**^MAF – minor allele frequency in 2504 individuals from 1000 Genomes project phase 3 release.
